# *‘We just don’t have this in us…’*: Understanding factors behind low levels of physical activity in South Asian immigrants in Metro-Vancouver, Canada

**DOI:** 10.1371/journal.pone.0273266

**Published:** 2022-08-25

**Authors:** Bushra Mahmood, Susan Cox, Maureen C. Ashe, Lindsay Nettlefold, Neha Deo, Joseph H. Puyat, Tricia S. Tang

**Affiliations:** 1 Faculty of Medicine University of British Columbia, Vancouver, British Columbia, Canada; 2 The W. Maurice Young Centre for Applied Ethics, University of British Columbia, Vancouver, British Columbia, Canada; 3 School of Population and Public Health, University of British Columbia, Vancouver, British Columbia, Canada; 4 Centre for Hip Health and Mobility, University of British Columbia, Vancouver, British Columbia, Canada; 5 Department of Family Practice, University of British Columbia, Vancouver, British Columbia, Canada; 6 Mayo Clinic, Alix School of Medicine, Rochester, Minnesota, United States of America; 7 Centre for Health Evaluation & Outcome Sciences, Saint Paul’s Hospital, Vancouver, British Columbia, Canada; Universiti Malaya, MALAYSIA

## Abstract

**Background:**

South Asian immigrants in western countries are at a high risk for metabolic syndrome and associated chronic disease. While a physically active lifestyle is crucial in decreasing this risk, physical activity (PA) levels among this group remain low. The objectives of this study were to explore social and cultural factors that influence PA behavior, investigate how immigration process intersects with PA behaviors to influence PA levels and to engage community in a discussion about what can be done to increase PA in the South Asian community.

**Methods:**

For this qualitative study, we conducted four Focus Group Discussions (FGDs) among a subset of participants who were part of a larger study. FGD data was coded and analysed using directed content analysis to identify key categories.

**Results:**

Participants expressed a range of opinions, attitudes and beliefs about PA. Most believed they were sufficiently active. Women talked about restrictive social and cultural norms that discouraged uptake of exercise. Post-immigration levels of PA were low due to change in type of work and added responsibilities.

**Conclusion:**

Health promoters need to consider social, cultural, and structural contexts when exploring possible behavior change interventions for South Asian immigrants.

## 1. Introduction

South Asians in Canada, who represent one-quarter (25%) of the visible minority population and 5.6% of the entire population [1], are less physically active than white Canadians [2, 3]. They also have a substantially higher prevalence and incidence of chronic disease (including cardiovascular disease, type 2 diabetes and hypertension) and other chronic disease risk factors like lower high-density lipoprotein cholesterol levels and a higher percentage of body fat [2]. According to the population level Canadian Community Health Survey (2000–01, 2003), compared with seven other ethnic groups, South Asians have the lowest prevalence of moderate intensity physical activity (PA) [3]. Various reasons have been documented for low levels of PA among South Asian immigrants. An overall lack of emphasis on PA and its association with health in South Asian culture makes it difficult to adopt an exercise regimen even when advised by doctors [4]. Prioritizing family time over other activities has also been frequently reported in this community [4, 5]. Furthermore, South Asian women also bear a greater responsibility for food preparation and raising children leaving little time or energy for any leisure time PA. Finally, low levels of PA have also been attributed to lack of awareness regarding the duration or intensity of PA that confers a health advantage, significant structural barriers like lack of women-only facilities and provisions for seniors, language barriers, and lack of confidence in participating in exercise programs [4, 6–9].

Promoting culturally sensitive PA compatible with the community’s needs is integral to the success of a PA intervention [10]. For this, an in-depth understanding of internal and external motivators, personal values and the social context of South Asians’ day to day life is crucial [9]. Previous studies show immigration status to be associated with physical activity behavior where, mostly recent immigrants (≤ 9 yrs) have been less physically active than older immigrants (≥ 10 yrs) [11, 12]. Currently, one of the largest South Asian communities in Canada exists in the Metro-Vancouver area, where the majority of the community members are from the Indian province of Punjab [13]. Given our lack of knowledge regarding this community’s attitudes, beliefs and conceptualization of PA, the aims of this study were to: 1) understand how PA is understood, perceived and conceptualized and explore social and cultural factors that influence PA behavior 2) investigate how the immigration status intersects with PA behaviors to influence PA levels and 3) engage the community in a discussion about strategies to increase PA in the South Asian community.

## 2. Methods

### 2.1 Participants and recruitment

Participants in this qualitative study were a subset of a larger cohort study (n = 425) recruited from Hindu temples and Gurdwaras (Sikh places of worship) in Metro-Vancouver, Canada. Participants in the larger cohort study were South Asian adults (≥21 years of age), living in Metro Vancouver, Canada, with no known previous diagnosis of diabetes; able to speak Punjabi and/or English and identified at an increased risk of diabetes based on the 7-item American Diabetes Association diabetes risk test [14]. A subset of 100 participants was recruited for a seven-day study where their PA levels and sedentary time were measured by an accelerometer. Focus Group Discussions (FGDs) participants were recruited from the subset of those 100 participants who took part in the accelerometer study.

We contacted potential participants via phone and explained the purpose of the FGDs, including their time commitment. According to Guest et al., three to six groups (with six to eight individuals) are sufficient to capture 90% of themes in a homogenous study population using a semi-structured discussion guide [15]. We continued phone calls to elicit participation until a convenience sample of 20–30 participants was secured (average of five to seven persons per focus group for a total of four focus groups) [15].

### 2.2 Data collection

#### Demographic data

We include in this study relevant socio-demographic data self- reported by participants in the larger cohort such as age (years), sex, years since immigration to Canada, education level, employment status, and total household income (CAD$).

#### Focus group discussions

Focus group methodology was selected over individual interviews for its potential to prompt a richer discussion through the interaction between participants [16]. FGDs were held between May 2019 and November 2019 and lasted from 60 to 90 minutes. All FGDs were held at Gurdwaras and moderated by researcher BM. Discussions were held in Punjabi and were guided by an interview guide based on approximately 8 to 10 questions with additional probing questions. Some of the questions on the guide aimed to explore how participants conceptualized PA, perceived community/cultural or family-based influences in the adoption of a physically active lifestyle, how being an immigrant in Canada had impacted their PA levels and how PA levels could be increased. All participants gave their written and signed consent for this study. The discussions were digitally recorded with the permission of all participants.

### 2.3 Approach to inquiry and theoretical frameworks

#### Paradigm of naturalistic inquiry

This research was guided by the interpretative framework of Social Constructivism which posits that there are multiple constructions of reality and that one’s perspective and experience is central to all definitions of reality [17]. Within this research we explored PA experiences of South Asian immigrants to Canada as the immigration experience shapes behaviors and experiences that in turn may impact health behaviors. Therefore, it is important to gain a clear understanding of the shared experiences of this group in order to better understand their PA patterns and observe how cultural norms and immigrant status shape their views and behaviors around PA.

#### Behavior change models

**Fig 1:**
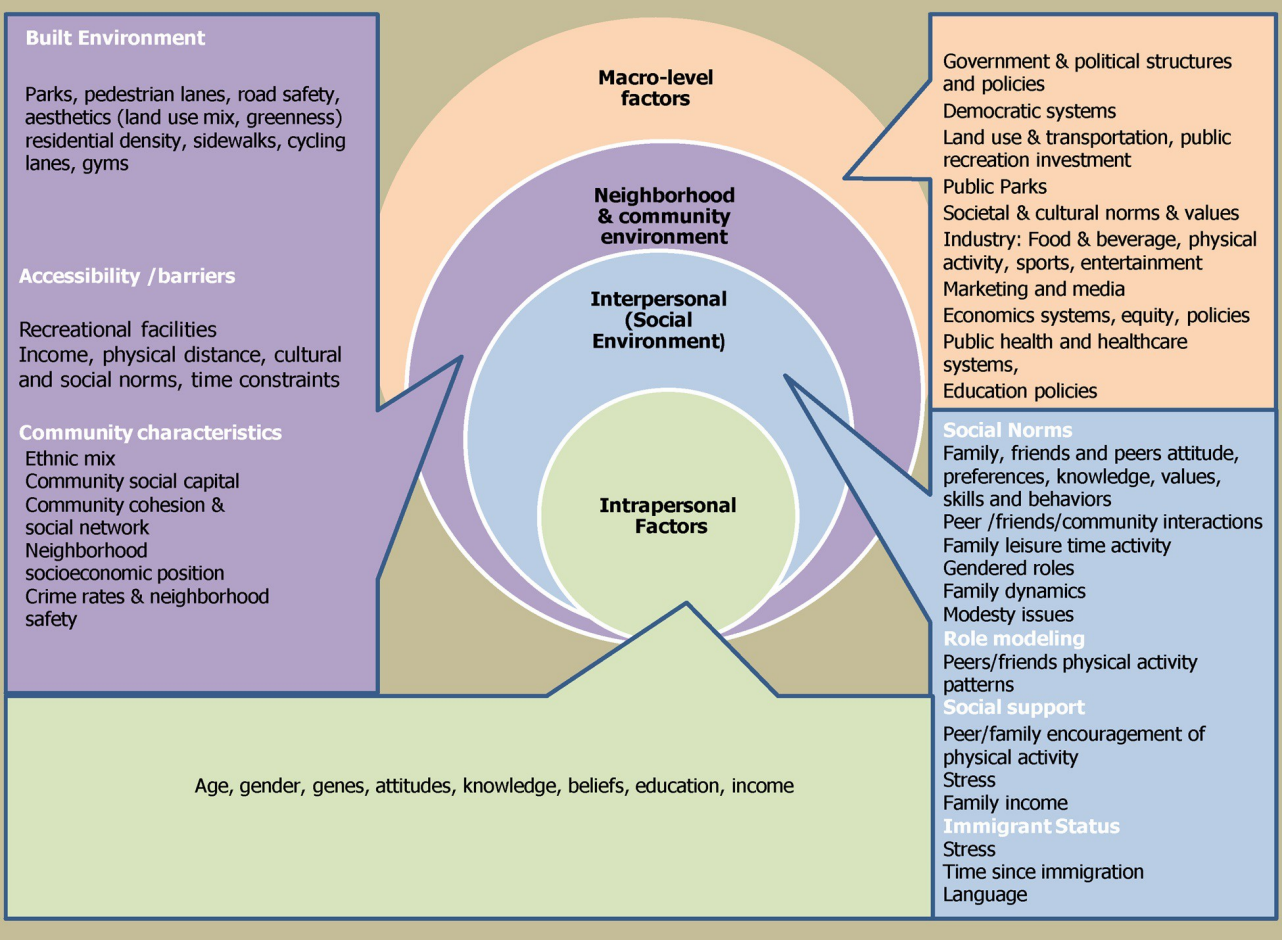
Social ecological model *(Adapted from Storey*, *M and K*.*K Davison*, *2008)*.

Previous studies have used behavior change models to explore personal level influences (knowledge, attitudes, beliefs) and inter-sectoral (family, community, organizational) influences that may impact PA or other health related behaviors in South Asians [6, 18, 19]. We applied the Social Ecological Model (SEM) (Fig 1) and the Health Belief Model to our study to understand factors that influence lifestyle behaviors like Physical activity. These models provided a framework for our FGD’s question guide (Table 1). Under the SEM, our questions explored the impact of cultural and social influences like gender norms, family dynamics, support and encouragement from the family to take time out for PA. Under the Health Belief Model, we explored individual level factors like knowledge regarding PA guidelines (What does PA mean to you? How much PA/day is considered sufficient?), and attitude and beliefs regarding importance of regular PA (How physically active are you?) and its association with chronic disease. We also investigated the effects immigration related stress on PA behavior as well as how immigration had influenced levels of PA.

**Table 1:** Key questions used to lead focus group discussions.

• What does the term physical activity mean to you?
• How much physical activity is required to stay fit and healthy?
• Family level factors that may influence physical activity
• Social and cultural norms that may influence physical activity
• Factors influencing physical activity levels before and after immigration to Canada
• Community discussions, level of awareness regarding physical activity
• What can we do as a community to increase physical activity levels?

#### Social Ecological Model (SEM)

A Social Ecological framework identifies key issues related to knowledge, attitude, and socio-cultural and religious factors that may inhibit or facilitate PA among the South Asian community. According to the SEM, behavior affects and is affected by multiple levels of influence and individual behavior shapes and is shaped by the social environment (reciprocal causation) [20]. This model considers the dynamic inter-relations and the complex inter-play between individual, family, and community level factors as well as macro-level influences such as policy that may directly or indirectly impact health behavior [21, 22].

#### Health belief model

The Health Belief Model provides an explanation of individual level factors in the context of individual level knowledge, beliefs, and attitudes. The core assumption of this model is that individuals will take a health-related action if, they believe it threatens health (perceived threat) and has serious consequences (perceived severity), they know that taking an action can avoid that threat and they have control or faith in their abilities (self-efficacy) to take that action [23].

### 2.4 Data analysis

We applied a directed content analysis which is a reflective, non-linear process and involves identifying and condensing meaning units, coding, categorizing and creating themes [24]. This approach is usually applied when prior research exists about the topic. In this case there is a body of literature on conceptualization of PA and perception of barriers and facilitators in adoption of an active lifestyle among South Asians [9, 25]. Hsieh and Shannon have developed two strategies for conducting directed qualitative content analysis [26]. The first strategy is based on reading textual data and highlighting those parts of the text that appear to be related to the predetermined codes dictated by a theory or prior research findings. Next, the highlighted texts are coded using the predetermined codes. For the second strategy, the coding process is initiated without primarily highlighting the text. In both analysis strategies, the qualitative researcher is supposed to return to the text and perform reanalysis after the initial coding process [26]. This second strategy provides an opportunity for recognising missing texts related to the predetermined codes and also newly emerged ones. It also enhances the trustworthiness of findings [27].

Based on past literature, some key themes were developed to lead the FGDs (Table 1). We started with how the participants conceptualized PA, what meaning they accorded to the term ‘physical activity’, what their level of awareness was regarding the PA guidelines and their self-perceived activity levels. We then delved into social and cultural influences that are known to impact PA behavior. Since all our participants were first generation immigrants, we also wanted to explore how immigration had affected their PA behavior. Finally, with an intention to engaging our participants in a dialogue about what, as a community could be done to increase activity levels, we focused on what type of conversations, if any, about PA were currently taking place within the South Asian community and how these could be used to develop effective interventions.

Audio files were transcribed by a team of trained bi-lingual researchers and coding was conducted by two authors (BM and ND) independently. In case of a potential conflict in coding, the decision was adjudicated by a third coder (TT). Transcripts were read and re-read to obtain an overall sense and an understanding of thoughts and ideas shared. Using existing theory or prior research, we began by identifying key themes or concepts and used these to create initial coding categories as suggested by Erlingsson et. al. and Hsieh [26, 28]. Two coders independently highlighted the text line by line and condensed the text into smaller parts or meaning units that were further condensed and coded using initial categories in the next step. These codes were then grouped under various categories which were then coalesced to form themes [28]. Any data that did not fall under the original coding categories were identified and analyzed to determine if they were to be classified as a new category [26]. A final coding matrix was developed and all data reviewed again and coded based on the final categories and subcategories.

Research objectives were clearly articulated to all participants. The consent form clearly outlined research objectives, rights and obligations of the participants and the voluntary nature of their participation. Participants were informed as to how the data will be collected (audio-taped) and used for research (transcribed). Consent forms were read in Punjabi/English as required and all participants were asked to sign if they agreed to participate.

The Clinical Research Ethics Board of the University of British Columbia (H13-00189) and Fraser Health Research Ethics Board (FHREB 2013–030) approved this study.

## 3. Results

A total of four FGDs, with 22 participants were held with the first one being all female, next two being mixed-sex and the last one being all male. By our fourth FGD, we decided we had reached saturation as the number of new themes dropped significantly and ideas were being repeated.

Demographic characteristics of our FGD sample are presented in Table 2.

**Table 2:** Focus group discussion participants’ characteristics.

**Age**			
	Mean (SD)	61.6 (10.2)	
	Median (IQR)	63 (13)	
	Range	40–79	
**Years since immigration**			
	Mean (SD)	25.5 (12.2)	
	Median (IQR)	23 (26)	
	Range	8–44	
**Sex**		**N**	**%**
	Women	12	55
**Education**			
	<High School	11	52
	High School	6	29
	>High School	4	19
**Income (CAD$)**			
	<$20,000	5	26
	20,000–49,999	10	53
	≥50,000	4	21
**Employment Status**			
	Currently working	7	33
	Not working	14	67

**Impact of group dynamics.** Dynamics of the focus groups was influenced significantly by the type of group (all male/female vs mixed-sex). Socio-cultural factors seemed to influence the nature of engagement and interaction within groups. Specifically, in the all-female group, women appeared to be more vocal in the absence of men, to talk about how social and cultural norms restricted their physical mobility and educational attainment thus limiting the realization of their full potential. Women talked about demanding husbands expecting chef-style meals, their inhibition in joining gyms, taking up swimming etc. due to fear of being seen in sportswear by South Asian community members. These views were reinforced by sharing of common experiences as discussed below. Similarly, men (all male group) may have been more vocal about acknowledging how hard life was for women in Canada than they may have been in the presence of women.

### 3.1 What physical activity means to me

Our FGDs uncovered a range of opinions, beliefs and attitudes towards PA among our participants.

In the absence of an equivalent terminology for ‘physical activity’ in the Punjabi language, participants often used the word ‘exercise’ to describe their understanding of the term. Walking, going to the gym, running, yoga, playing sports like soccer were some of the activities that constituted ‘physical activity’. Most participants understood the overall health benefits of keeping active. Importance of stepping out in ‘fresh air’, not being ‘lazy’ and ‘keep moving even if there is an injury’ were emphasized or else, it was stated, diseases like ‘blood pressure, diabetes’ could occur. Some of the factors that encouraged participants to be active were to ‘keep one free of disease’ and consequent dependence on ‘medication’ or ‘other family members’. Benefits of regular PA on mental health and digestive system were also mentioned. One female participant in the all-female-group had this to say:

*“If you are active*, *your mind stays active*. *Your health remains good*. *Food gets digested well*.*”*

For women, being physically active meant doing housework and taking care of grandchildren. There was, however, some disagreement- as we can see from the conversation that transpired between two participants (in all-female group) whether housework could be considered PA. According to one female:

*“The cooking that we do*, *that’s also exercise*. *When we stir the vegetables and when we are working in the garden*, *we bend*, *have some cultivation at home–or when we kneel to cut grass–and all this*, *although is not too much*, *but is exercise nevertheless”*

Another female disagreeing with the above had this to say:

*“No*, *no*, *no–not this*. *We need to take time out for ourselves*, *decide a place or a spot where you will go–for half an hour or an hour or 20 minutes–that’s what you should do*. *House cooking or work–that is not exercise*. *I don’t believe that is exercise*.*”*

Additionally, sitting all the time seemed to have a negative connotation and was viewed as being ‘lazy’. Many of our female participants (in mixed-sex as well as all-female groups) talked about getting ‘diseases’ if one was ‘lazy’ and not physically active. Moreover, being too sedentary was, in all seriousness, considered a risk for developing ‘laziness germs’ which, it was believed would create a never ending cycle of perpetual laziness:

*“If you don’t exercise and are lazy*, *then all kinds of diseases*, *especially diabetes*…*”*

*“If we are lazy and keep sitting then we will develop laziness germs*. *And they will weigh us down more*. *We won’t be able to wake up- will feel like sitting all the time*.*”*

*“…Husband will say you are having tea–sitting lazy in front of the TV*…*”*

### 3.2 How much physical activity is sufficient?

We asked the participants about duration and frequency of PA. Only one out of the six participants in the all-female group stated that 30 minutes of PA per day was sufficient while the rest believed there was no set time and that housework and taking care of the children kept them busy all day. According to one participant, (all-female group):

*“Fifteen minutes*–*we do that at least*–*barely [laughs]”*

According to another female participant (mixed-sex group):

*“There’s so much to do at home all day–cut the grass*, *give water [to plants]*, *do stuff inside or outside*, *wash the clothes*. *It’s not that we’re sitting on the sofa or on the chairs*. *Meaning at home–climb the stairs 10 times and come down the stairs meaning we…that’s good too*.*”*

The majority of participants in all-male and mixed-sex focus groups seemed to have a clearer idea about how much PA was needed—at least 30 to 60 (or even more) min/day. Male participants offered concrete ideas on how to break down prolonged sedentary time at work by taking 5 to 10-minute walking breaks to improve circulation. As one male participant (all-male group) suggested:

*“… Now see even if you are working two hours and you have 10 minutes even*, *do it then*. *After one hour*, *then do it again for 10 minutes*. *Then together you can add it to half an hour*.*”*

While only a few participants were employed, occupation related PA was also considered a source of ‘exercise’ as a female participant (all-female group) talked about the physically intensive job she had where she stands for almost 8 hours and sorts out clothes. Walking appeared to be the most popular exercise among the participants–especially those relatively older in age. As one male participant (mixed-sex group) stated:

*“See dear*, *for 100 health problems*, *there’s one treatment*. *Meaning among 100 illnesses*, *there’s one treatment and that treatment is wholly walking*. *Walking is a very good thing–it’s a very wise thing*.*”*

Men who were older (≥60 years) mostly identified walking to and from the Gurdwara every day as a significant source of their PA while the younger men (between 43 to<60 years), talked about playing sports, running and going to the gym. One female participant (mixed-sex group) pointed out that going to the gym was even better but this did not seem a popular trend in the South Asian community.

### 3.3 Perceived physical activity levels and factors that may influence physical activity

When asked about the self-perceived levels of PA, the majority of participants believed they were ‘sufficiently active’. As one female participant (mixed-sex group) stated:

*“I do very much—a lot of exercise*.*”*

Looking younger than one’s age was viewed as being synonymous with physical fitness. Several participants also believed they were much more active and fit than the younger generations. One male participant (mixed-sex group) spoke about how his children who were over-weight, could not do as much work as him. For women, since house-work kept them busy all day, they felt they were sufficiently physically active. As one female participant (mixed-sex group) noted:

*“I*, *actually in the morning go for a walk for half an hour*, *and for the rest [of the time]*, *for the children—there’s grandchildren–after getting them ready*, *then after making them food and feeding them*, *then going and dropping them off at school*, *and then going and giving lunch–like that–then the whole day–then I need to clean the house and do other work…then yes*, *keep moving around*. *The household chores just don’t end for women*.*”*

Participants realized there were several other factors that influenced one’s level of PA such as physiological changes with age, stress levels and work demands, as a male participant (mixed-sex group) stated:

“*The rest depending on your age*, *the work you do*, *activity and exercise are always changing*. *As the body changes*, *along with that time and stress*, *how much workload we have it decreases*. *There is no definite formula*.*”*

A female participant (mixed-sex group) talked about how depression had affected her physical mobility:

*“Mentally… we have to think mentally too–how your mental capacity is*. *Yeah*.

*Yeah*. *Depression medications…*. *As X was saying*, *sometimes I even have arthritis*. *A depressed person is in pain*. *Sometimes*, *one can’t even get up during the day*. *So how you feel*, *I am saying*, *somebody motivates you…”*

Participants who were older (≥70) mostly talked about health conditions and age restrictions on their ability to be more active, demonstrating some stretching exercises they did either in bed early in the morning or while standing up. According to one female (all-female group):

*“Now that we are in this age*, *we cannot do a whole lot of exercise when we are this old*. *We can walk a little–can just do light exercise*. *One should exercise according to one’s age*, *not putting your body under too much pressure*.*”*

Another female (mixed-sex group) mentioning her health condition which negatively affected her physical mobility had this to say:

*“My son said to me “Go for walk*, *go for gym*, *go and gym*, *why you sleep*.*” I said—I was very upset*, *right*. *I have a problem in my feet and hands*…*”*

According to South Asian women who were older, poor weather conditions also seemed to hamper PA levels, more so for South Asians than people of other ethnic groups. People who were older (≥70) and had breathing issues like asthma were particularly affected by the cold. Danger of slipping during rainy weather was also a constant risk. However, apart from the above deterrents, there was a general recognition that PA was not prioritized in the South Asian community. As one female participant (all-female group) admitted:

*“But we don’t do as much as is required–is necessary for our health–we don’t do that much*.*”*

She believed it was not so much the extraneous factors to be blamed but had more to do with women themselves.

*“And like what they said earlier- sometimes there is no time- but we need to take time out for ourselves*. *We take time for our children and for other tasks*. *For watching TV and other stuff- well*, *entertainment is also important but for exercise*, *if not too much then at least half an hour*, *if we can take out every day*.*”*

She continued:

*“We have [driving] licenses*, *we also have cars at home… we blame others…that someone doesn’t let us go out*. *I don’t feel that everyone has this problem*. *We should go to the gym*, *we should go swimming as well*, *or we should go to some lake*, *and sit*, *and spend time there*.*” (Mixed-sex group)*.

According to another female participant in the all-female group, the need was to ‘awaken our women’ [make them aware].

### 3.4 Gender roles, family dynamics

South Asian women have been identified as being at a particularly high risk for physical inactivity. Some participants talked about challenges women faced trying to live up to certain gender norms. A female participant (all-female group) who was currently employed, identified cooking for her husband as the biggest hurdle towards her low levels of PA:

*“For me it’s my husband’s cooking*. *[laughs]*. *I have to make his lunch*. *If it was not for that*, *I could go anywhere… He does not like to eat from outside…food of my house*, *cooked by me- that is how it should be for him*. *And for that*, *I need two hours for sure*, *for cooking*. *It’s not that you just do it like this and give it–no*. *It should be good*, *perfect food*.*”*

Men also seemed to acknowledge that it was tougher for women as one male participant in the all- male group stated:

“*For them it’s a big problem because they now go to work and also do housework*. *For them it’s a very hard job*. *For them exercise…if they need anything it is help*…*”*

Another male participant (all-male group) agreeing to the above added:

*“Especially in these countries–it’s different in India and Pakistan*. *There is a person for washing dishes*, *a person for washing clothes*. *Person for cleaning*, *different person for cutting vegetables*. *Over here [in Canada] you have to do everything yourself*. *It’s a hard life for ladies*.*”*

While the majority of the female participants talked about being busy with household chores and childcare, they viewed these activities as their biggest source of PA and hence, not an obstacle towards being able to take time out for PA. As one female participant (all-female group) noted housework, instead of being a ‘barrier’ was actually refreshing:

*“Yes–but I am able to do all the housework- whatever there is*. *Well that also refreshes your mind- we go out as well–going to the store*, *the mind gets refreshed*. *All our system- we get energy [body feels energized]*.*”*

It appeared that there was unwritten expectation that women who were older and living in multi-generation homes would contribute towards taking care of the grand children as one female (all-female group) had this to say:

“…*And if we are not even going to take care of grandchildren then*…. *[what else are we going to do]*?*”*

Women seemed to take pride that they could still manage to take care of the grandchildren and run the house–despite their advanced age, as one woman in the all-female group noted:

*“But despite that*, *according to my age*, *I am about 74*, *exercise is very good but I do a lot of housework too*. *All of it–I mean–all the kitchen- I take care of it*.*”*

We inquired about family influences that may encourage or hinder PA. For the most part, women who were older reported being encouraged by their children to be active, exercise and take time out for themselves. According to one female participant, (mixed-sex group):

*“I get full support–from the children as well–they say to us–they make a schedule for us and tell us everything like “do this*, *you should eat this*.*”*

One male participant (mixed-sex group) talked about how he encouraged his mother and her friends to start walking rather than sitting in the park and talking. Unlike female participants, male participants noted they had no family restrictions or responsibilities that would constrain them from being active–especially now with the children all grown up. Some also talked about helping out at home with house chores and stated there should be no ‘shame’ in helping your wife.

### 3.5 Cultural influences

In South Asia there is a strong culture of respect towards older people in general, including one’s parents which is often manifested in serving them at all times. As such, adult children may unknowingly end up discouraging their elderly parents from performing light PA. We probed this question with participants of relatively older age, to explore if they had encountered similar situations from their children/daughter-in-laws. While none of the participants experienced this, a male participant (mixed-sex group) acknowledged that restricting elder parents from working around the house was an expression of ‘respect’.

*“… My mom who is 86 years old*, *she would do work and all of us would get angry–the 6 of us–the 3 brothers and the 3 wives–like ‘mother*, *you should sit down*, *if someone comes*, *they will say that no one lets the old woman sit*.*’ My mother would tell us ‘Son*, *I’m not doing this because of you*, *I’m doing this for my health–I do it for that*. *The day I sit*, *I will remain sitting*.*’ This is why she did work*.*”*

He also noted that in India, they do not let elder parents work because people ‘talk’. Participants agreed with the importance of working well into your old age as one female participant (mixed-sex group) concluded:

“*This means that like if we drop everything and say that …our sons and daughter-in-laws are here now*, *let’s sit–this is all wrong*. *As long as you can keep it going*, *keep it going…”*

Women appeared to have been negatively impacted by some traditional cultural practices which influenced their lifestyle behaviors. Several female participants (in all-female group) attributed low levels of PA in South Asian women to the way they were brought up in India–the overall cultural influences that inhibited young girls from being physically mobile or getting good education in cities. As one participant claimed:

*“They [parents] would stop us from everything…even the educated ones*, *among the friends*, *would also be afraid- that girls should not do this thing–I am telling you the truth*.*”*

She went on to explain how lack of awareness about PA and restrictive cultural and social influences held them back from everything:

*“At that time*, *in the cities or village*, *there was no such education telling us we need to stay active*, *we need to live like this*, *girls need to do that- because women have always been held back*. *A girl cannot do this; a girl cannot do that*. *Cannot go there…”*

Her response to why South Asian immigrant women in a country like Canada (where, unlike in India, there are no safety concerns and most have access to parks and safe neighborhoods to walk in) are still not active enough, was:

*“At that time girls would only do JVD [referring to some educational degree]*. *At that time I did not get admission close by [in school/college]*. *My family did not send me far–that we are not going to send her away -*. *These things remain in your heart*. *So over here*, *you are so old now*, *have seen everything*, *but still we have to ask our husbands for everything and support them*. *That’s a habit now that was developed then*.*”*

South Asian culture was also perceived as being inhibitive towards women who wanted to initiate an exercise regimen. One female participant (all female-group) had this to say:

*“The other thing is that*, *now it is a little bit different–in our community*, *when a woman goes out alone…to start doing that*, *is very difficult*. *Because in our community*, *people are like*: *‘huh*, *she goes to the gym…now she must be dressing up like that’*. *They have not seen her at the gym but …you can wear whatever you want to*, *but they will say*, *she is dressing up like white people now*.*”*

Modesty and fear of what the community members may think (especially South Asian men) appeared to be a strong factor in discouraging women from accessing gyms and taking up activities like swimming–as one female (all-female group) had this to say:

*“If we say we go for a swim then they say ‘she must be wearing swimsuits*, *there must be south Asian men there’*. *Even when we ourselves begin [exercise regimen] we feel the same way…that there will be South Asian men and we are wearing such clothes*, *how will it look*. *Now I am in Burnaby–I go*. *I would feel the same way too*. *I got a suit which covered me up till here [shows] minimum*, *at least this long–right*? *With smaller suits I was like there may be someone from our community…because I have never been out dressed like this before*. *But once I started going there*, *I felt fine*. *The advantage I had was that over there is that our community people do not go*. *There are none–over here*, *in Surrey*, *there are more*.*”*

She also noted that if South Asian men were accompanied by their wife, it made them feel more comfortable to be in the gym by themselves. This was mostly because being with one’s wife, would make a man less likely to stare or be disrespectful at another South Asian woman who was by herself.

Participation in active sports is not very popular among South Asian immigrants. One male participant (all-male group), talked about how his close friends and neighbors reacted to his interest in marathons upon arriving in Canada:

*“…My friends and pals and the neighboring- all around would say ‘Why are you taking a risk*, *raise your kids*. *You are in Canada*, *raise your kids*. *You will break your leg*, *where will you go*, *your kids will starve and die*.*’ And I am like why will they die*?*”*

### 3.6 Physical activity levels–before and after immigration to Canada

The majority of our participants came from villages in India and were from an agrarian background. While men had performed physically intensive work in the fields, tilling soil (with bulls), farming, and harvesting, women, other than cooking and household chores, also milked buffaloes, churned milk, and helped in other farm work.

As one female participant (mixed-sex group) put it:

*“We got so tired there…now we think it is time for us to rest in this country [Canada]*. *Now it’s our time to rest so let’s just sit and rest*. *But we don’t know that we are procrastinating*…*”*

PA was woven into the fabric of their life as farmers and there was no need to take extra time out for it. As one male participant (mixed-sex group) stated:

*“Because in India we had to do a lot of physical work like ploughing the fields…We did not need to walk or exercise over there*. *We would go to the fields*, *use a kae [an iron gadget to cut grass]* … *we would hold a kae and cut grass–how was the tummy going to bulge- if anything*, *it would go in*. *Because we would use kae*, *water the fields*, *used our hands to cut hay… milk buffaloes–there is no need for exercise over there–as our hands*, *arms and legs would be moving*.*”*

Upon immigration to Canada, most participants talked about working in mills while women raised the children. The work in the mills was very ‘hard’. Participants talked about life being very stressful as recent immigrants with no time to be more active when first arriving in Canada. When asked about PA level on arriving as recent immigrants, one female participant (mixed-sex group) responded:

*“What active*? *We didn’t even know about ourselves [were so busy] … we had to get established*. *The families who were left behind us in India from both sides [maternal*, *and in-laws]–we had to get them here too*, *and get them married*, *and get their letters [immigration paperwork]*. *Then*, *our own children*…*”*

Another male participant (all-male group) however noted that he became more active upon immigration as back in India, he was a student and had to sit and study for long hours since excelling in school was the top priority. Participants also talked about an overall increase in level of awareness around PA as one male participant (mixed-sex group) noted:

*“At that time*, *the mindset was different*. *Now the awareness in the media has changed the mindset towards ‘activity is the best way if you want to live’*.*”*

Several other participants also believed there was a lot of knowledge around a healthy lifestyle as compared to before. According to one male participant (all-male group):

“*… Even if a person these days does not exercise*, *I notice that he is conscious about what he is eating or trying to control that—and other than food*, *their attention is also towards exercising*. *And they are also conscious about after eating*, *how much they need to walk*.*”*

The majority of participants also thought that now they were more active than their peers back home in India. According to one female participant (mixed-sex group):

*“No*. *No they [friends back in India] are not active*. *Their daughter-in-laws move in*, *and they become dependent upon them*.*”*

Participants talked about cheap labor in India and dependence of people on domestic help to cook, do household chores, and drive. Even the farmlands, according to the participants, had now been handed over to contractors and much work in the fields had been mechanized. As one female participant (all female-group) noted:

*“Compared to here [Canada]*, *over there [India]*, *there is no comparison*. *Families over there*, *they will be waiting for the servants to come and make tea for them*. *I was there with my niece for two months*. *And when I would go to the kitchen to do some work*, *she would be like no*, *you sit massi [Aunt]*. *He [servant] will come and make tea*.”

Another female participant (all female-group) added:

*“No- now at this time*, *ladies my age- they are like now we have blood pressure*, *our knees hurt*, *now we have this [health condition]*. *They don’t do any work*. *Before they would be churning milk*. *Now there is nothing they do*. *Even the ones who are not very financially well off*, *will have at least two helpers- one to clean the floors and one to wash clothes*.*”*

Comments from many in the group supported the belief that the health of their peers was deteriorating at a faster pace attributing this difference to a sedentary lifestyle. According to one female participant (all-female group):

*“…I have friends*. *Wahi Guru–we are all sitting here—one walks with a er… what you call it … wheelchair [walker]*. *Another poor friend- her knees hurt too just like me–she is my age and is sitting*. *One passed away*. *Whenever I go*, *all my nieces say ‘you are the healthiest compared to them all’*. *My knees have just now started hurting a little*. *But it’s Gurus blessing otherwise I am fine*.*”*

There was a tendency to compare South Asians with other ethnic groups and acknowledge some differences that existed regarding lifestyle. For instance, one female participant (mixed-sex group), who had been raised in Malaysia, noted that while labor and domestic help was cheap in Malaysia too, however, some of the other ethnic groups that she had lived with did not rely so heavily upon domestic help as people did in India.

*“Because no*, *but other races are active*. *The Chinese*, *other races*. *They also have servants and everything but they are very active*. *Illnesses depend on their health*. *They are active*.*”*

### 3.7 Within-community discussions surrounding physical activity

According to our participants, there were very few discussions around the importance of being physically active at the community level. Gurdwaras, where the community members met regularly, played little role in providing a platform for discussions around health and lifestyle.

One female participant (all-female group) mentioned a club she attended on a monthly basis where they shared health tips and invited health experts but for the most part, as one male participant put it, the conversations at most community gatherings were limited to ‘community gossip’. Another male participant however (mixed-sex group), differed with the views above stating that he had noticed a change in awareness from when he first arrived in Canada as an immigrant. Also, while growing up in India, he noted he had never seen women go out for a walk but now he would see big groups of women going out for a walk together, which, he believed was a welcome change.

### 3.8 What can we as a community do to increase our physical activity?

Participants expressed a need for face to face, question and answer format with the health care professionals. One female participant (all-female group) felt there was a need to educate the community. Another female participant (all-female group) highlighted the role men can play in promoting PA among women.

*“Yes- raise awareness among the people*. *Not just women but men as well*. *As when the men become aware*, *because sometimes they are active*, *they don’t have a lot of chores to do at home*. *They come home from work*, *they are idle*, *they go out*. *For women*, *housework totally ties them down*. *They cannot go out*. *And the man knows it too that my wife too needs to stay healthy so he will try and help her get out of the house*. *That help should be there–the women need to be helped–they need to take out so much time for themselves*. *If no one helps her*, *then she is going to be stuck there*.*”*

She continued with the following advice:

*“All in all*, *seminars are very important*, *in which there shouldn’t be anything extra said–[the seminar] should discuss food and health-related topics*. *Don’t just give us books to take home–we don’t understand them/get anything out of them*.*”*

Several participants talked about educating and raising awareness in the community about leading an active lifestyle. One male participant (all-male group) talked about a weekly dial-in radio program on the Punjabi channel in the past where a doctor was available to answer any health-related questions.

“*What doctor tells you vs what you read*, *we don’t have time to read*. *The knowledge you get from listening–I get more knowledge from listening–as there is no time to read and listening is easier–it makes sense to me*. *What he [the doctor] tells you–you follow that*.*”*

Some participants were of the view that there was no dearth of education. Everyone understood pretty well as to why it was important to take care of our diet and exercise regularly as one male participant noted (mixed-sex group):

*“There is a lot of awareness here*. *Everything is available on the TV and newspapers*. *A lot of programs air*, *a lot*. *Now it’s up to everyone*. *I say everyone should exercise*. *It is a good thing*. *And how ever much one can do*, *they should*.*”*

Another male participant in (all-male group) put it this way:

*“…the media [in Canada] informed us what is happening*… *everything*, *that is when we started to change ourselves*. *My whole life*, *I never ran or exercised*, *no … I had not done an activity because BSc science is really hard there [in India]*. *And*, *when I had come [to Canada] I was 50 years old*, *that is when I*… *I joined…our …gym*. *That’s when I did gym and that’s when I started running*.*”*

### 3.9 Recent immigrants–at greater risk for sedentary lifestyle and unhealthy diet

During the discussions, some participants identified newly arriving immigrants and their children from India as a truly high-risk group for sedentary lifestyle and unhealthy diet–which, unlike them, came financially better off and with no awareness regarding health-related issues. As one male participant (mixed-sex group) noted:

*“Children that are born here*, *they do not like Punjabi restaurants*. *Eating sweets or other foods–they do not do so*. *But the main problem is arising from those that are coming from back East*. *The children coming from India and Pakistan*. *New immigrants*. *Because their parents attend 3 marriage ceremonies a day*. *They attend three marriages and how much they must eat*! *They [the children] carry this in their genes*…*”*

He continued with the following conclusion:

*“So*, *it doesn’t matter how much we try to tell them or educate them*, *the genes will be suppressed momentarily but then surface ultimately*.*”*

## 4. Discussion

This study aimed to understand how physical activity was perceived and conceptualized by the South Asian community, how socio-cultural influences and participant’s immigration status intersect to impact this lifestyle behavior and finally, what the community members believe can be done to help the community members become more active. We were able to identify some unique, often intersecting factors that may help explain low levels of PA among South Asians.

Variation in how PA was explained or defined shows each individual had her/his own concept of what PA meant to them and not all of these concepts were congruent with that of health experts. Similar to other UK-based studies where (relatively younger) South Asian women (mean age 40 and 52 years) attributed most of their activity to housework (cleaning and cooking) and childcare [6, 29], our female participants also shared this view. However, many of these chores do not meet the definition of moderate intensity PA, and thus, may explain the incongruence between self-reported and accelerometer-measured moderate to vigorous PA (MVPA) levels in some mixed-methods studies [6, 29]. This demonstrates that women did not understand MVPA intensities. Similar to other studies with relatively older participants, [4, 30], age and other co-morbidities played a predominant role in what type of activity participants could engage in. Some older participants talked about being limited by ‘weak’ bones, asthma that became worse in the cold, painful knees and fear of falling while performing PA- especially in the rain. A few women who were older and claimed they were ‘sufficiently active’, demonstrated some of the exercises they did every morning -some before getting out of bed and others standing in the kitchen while cooking, but these were mostly stretches and did not meet the required intensity level to be considered moderate PA.

Although the majority of the participants were generally aware of the overall health benefits of regular PA, understanding of the actual levels/intensity of PA required to gain health benefits was limited. While LPA has its advantages, this finding, also reported in other studies [6, 29, 31] underscores the importance of clearly communicating the concept of activity ‘intensity’ to this group so that they know what activities qualify as moderately intensive and will yield health benefits. For instance, vacuuming, washing windows, sweeping thoroughly, are some of the household activities that have been shown to be conducted at moderate intensity (3–6 METS) [32]. However, since converting sedentary time into LPA seems to be a more achievable goal, the health benefits conferred by LPA should not be under-estimated. Studies have found an association between LPA and more beneficial plasma lipid profile as well as lower postprandial glucose and insulin levels in overweight/obese adults [33, 34] Additionally, health promotion programs need to tailor their interventions to the specific PA needs of different age-groups as there seems to be a gap regarding knowledge of what types of exercises can be safely performed by older people.

Unlike other studies [29], for the most part, the women in our sample did not frequently label housework as a ‘barrier’, as it was also viewed as their single biggest source of PA of which they took pride in. However, it is important to remember that most women in our study were older and retired and housework was not as intensive for them as it may be for younger women who may be working full time and also running the household and tending to family demands. A relatively younger woman who was also employed identified cooking demands as the biggest barriers to her taking up any PA.

While the majority of participants were able to quantify the amount of PA required on a daily basis (30 min brisk walk) and believed there was an adequate level of community awareness for being active, our all-female group was unable to state how much activity was recommended on a daily basis. This demonstrates that women who are older (and perhaps men as well) may not be cognizant of the amount of PA that would positively impact health which may partly account for low PA levels. Additionally, keeping in mind the physical limitations of older age as mentioned by many participants, the emphasis needs to be on ‘moving more’ as suggested by Canadian 24-Hour Movement Guidelines for Adults aged 65 and above according to which LPA including standing and lowering overall sedentary time also matters [35]. While some participants talked about and demonstrated some ‘stretching’ exercises which they said helped them with ‘stiff joints’, there was no conversation around resistance training and balance activities–especially pertinent for older participants who also talked about fear of losing balance and falling while exercising. This may be either because these topics were not brought up for discussion like some other issues deliberately touched upon in our conversations or because the participants lacked knowledge about benefits of these exercises. This is something that needs to be further explored in future research.

Low levels of PA seem to be partly explained by some social and cultural factors. Consistent with other studies [36], some participants (not from an agrarian background) reported that a greater emphasis on excelling in school discouraged participation in sports and related activities from early childhood. Fear of injury was noted as an additional deterrent. This is an indication of not only an overall low uptake of organized sports in the community as observed by previous research [37, 38] but also of unfounded fears around organized sports causing irreparable body harm thus incapacitating one to take care of his/her family.

Some gender-based restrictions also emerged during our discussions. Female participants talked about a reluctance to take up any regular PA regimen at the gym for the fear of being seen wearing active/swim wear and being labeled as becoming too ‘westernized’. Modesty as a concern has been observed by previous research too where it was mostly Muslim women from relatively more conservative backgrounds who talked about feeling uncomfortable wearing western sportswear [6, 7, 30]. Our findings underscore the need for providing access to older South Asian women of all religious denominations either all-women facilities or, spaces within facilities which are restricted to women only.

There is growing evidence on the positive effects of PA on mental health [39–41]. As reported in other studies (14), participants recognized the positive impact of PA on mental health. However, in our study, more female than male participants reported how ‘depression’ and ‘stress’ had reduced their desire to engage in any activity. South Asian women have been observed to be at a higher risk for depression resulting from social isolation [42]. Post-migration factors like loss of social status, social support, separation from family, difficulty integrating into a new culture, and lack of employment puts immigrants at a particularly high risk for stress and depression. First generation South Asian immigrants seem to be at a higher risk for untreated depression and stress given the stigma of mental health conditions within South Asian populations [43]. A study of older adult South Asians in Calgary found more than double the prevalence rate of mild depression (21%) compared to the national average (10%) [44]. The issue of stress among South Asian immigrants is poorly researched [7] and more understanding is required around how mental health affects health trajectories including adoption of lifestyle behaviors like PA. Furthermore, it is important to be aware of the competing priorities and complex challenges that recent immigrants face in trying to settle and integrate into the Canadian system as this has a direct impact on their mental health and thus it is within this context that health intervention strategies should be identified.

In India, health education is typically delivered by a doctor answering questions posed by community members. Not surprisingly, this method of health communication is what the majority of our participants described as the optimal approach. Involving the target community in developing culturally appropriate interventions appears to be important in their acceptability, delivery and uptake [45, 46]. Due consideration to the community’s preferred model of communication and learning has also been observed to improve intervention uptake [47]. This study highlights the need to tailor professional-led health interventions to meet the specific needs and preferences of the community—specifically regarding choice of an effective mode of communication of health messages.

Our participants were especially concerned about the current generation of recent immigrants who were not used to physically intensive work like their ancestors and had little knowledge regarding what constituted a healthy lifestyle and advised that this was the group that needed to be targeted by health practitioners. Indeed, a higher proportion of recent immigrants from visible minorities are inactive compared to established immigrants [11, 48, 49] and this underscores the need for targeting this high-risk group. Additionally, the role of Gurdwaras in becoming a platform for health was also raised as an option for health education communication. Participants believed Gurdwaras had a great potential to influence community thinking and needed to play a more substantial role in community health. A recent study examined the feasibility, acceptability and effectiveness of a mosque-based PA program for South Asian Muslim women in Canada. Apart from concluding the mosque-based program to be feasible and acceptable, at the end of the intervention, there was also a significant increase in self-efficacy scores and fewer women were classified as inactive [50]. Similar Gurdwara/temple-based PA interventions targeting South Asians in the USA have also been highly effective [51–53]. Collaborating with faith-based organizations with a captive audience thus has considerable potential and can be a financially viable avenue for health promotion campaigns.

### 4.1 Strengths and limitations

The study provides some unique insights into South Asians views, attitudes, and beliefs towards PA. Holding at least two same-sex focus groups had a clear benefit as both women and men talked about issues we feel they would not have felt comfortable discussing openly in the presence of the opposite sex. However, some limitations apply related to inadequate representation of the sub-groups within the South Asian diaspora and thus findings need to be interpreted with caution as they cannot be applied to all South Asians. Also, as we were not able to touch upon sedentary time, and its conceptualization among the South Asian community, we recommend that future studies explore this phenomenon adequately especially considering the relatively high sedentary time accumulated by the participants in our accelerometer-based study [54].

### 4.2 Conclusion and implications for policy and research

Findings from our study highlight the importance of considering social, cultural, and structural contexts when exploring possible behavior change interventions for South Asian immigrants. Similar to other studies, our data shows a strong presence of ‘collectivist’s influences’ like the family unit and the wider South Asian community and migration-related challenges [55, 56], the recognition of which is important in developing and implementing successful health promotion programs.

Additionally, it is important to recognize that this ethnic group is not homogenous as can be seen from diverse and sometimes conflicting findings from similar studies [57] thus cautioning health promoters against the danger of stereotyping the ethnic group. There are variations within the community members of experience and perspective regarding PA, as noted by other studies and these experiences and perspectives are not static as these vary according to heterogeneity within ethnic groups based on, for example, acculturation, geography, socioeconomic status, age, and gender [45]. These factors need to be considered for developing tailored, highly effective health promotion interventions.
